# Oil Bodies from Chia (*Salvia hispanica* L.) and Camelina (*Camelina sativa* L.) Seeds for Innovative Food Applications: Microstructure, Composition and Physical Stability

**DOI:** 10.3390/foods12010211

**Published:** 2023-01-03

**Authors:** Christelle Lopez, Hélène Sotin, Hanitra Rabesona, Bruno Novales, Jean-Michel Le Quéré, Marine Froissard, Jean-Denis Faure, Sylvain Guyot, Marc Anton

**Affiliations:** 1 INRAE, UR BIA, F-44316 Nantes, France; 2 INRAE, UR BIA, F-35653 Le Rheu, France; 3 INRAE, PROBE Research Infrastructure, BIBS Facility, F-44316 Nantes, France; 4 Université Paris-Saclay, INRAE, AgroParisTech, Institut Jean-Pierre Bourgin (IJPB), F-78000 Versailles, France

**Keywords:** lipid droplet, oil body, interface, membrane, natural oil-in-water emulsion, plant-based food

## Abstract

Exploring and deciphering the biodiversity of oil bodies (OBs) recovered from oilseeds are of growing interest in the preparation of sustainable, natural and healthy plant-based food products. This study focused on chia (*Salvia hispanica* L.) and camelina (*Camelina sativa* L.) seed OBs. A green refinery process including ultrasound to remove mucilage, aqueous extraction by grinding and centrifugation to recover OBs from the seeds was used. The microstructure, composition and physical stability of the OBs were examined. Confocal laser scanning microscopy images showed that chia and camelina seed OBs are spherical assemblies coated by a layer of phospholipids and proteins, which have been identified by gel electrophoresis. The mean diameters determined by laser light scattering measurements were 2.3 and 1.6 µm for chia and camelina seed OBs, respectively. The chia and camelina seed OBs were rich in lipids and other bioactive components with, respectively, 64% and 30% α-linolenic acid representing 70% and 53% of the total fatty acids in the *sn*-2 position of the triacylglycerols, 0.23% and 0.26% phospholipids, 3069 and 2674 mg/kg oil of β-sitosterol, and lipophilic antioxidants: 400 and 670 mg/kg oil of γ-tocopherol. Phenolic compounds were recovered from the aqueous extracts, such as rutin from camelina and caffeic acid from chia. Zeta-potential measurements showed changes from about −40 mV (pH 9) to values that were positive below the isoelectric points of pH 5.1 and 3.6 for chia and camelina seed OBs, respectively. Below pH 6.5, physical instability of the natural oil-in-water emulsions with aggregation and phase separation was found. This study will contribute to the development of innovative and sustainable food products based on natural oil-in-water emulsions containing chia and camelina seed OBs for their nutritional and health benefits.

## 1. Introduction

Consumers now indisputably understand that their health is strongly related to their diet. Clear evidence for this is their growing interest in natural and minimally processed foods as well as in sustainable agricultural and food practices. Foods providing an adequate nutrition including essential bioactive molecules are important elements in the prevention of many civilisation-related diseases such as diabetes, cardiovascular diseases and obesity. In recent years, there has been an increasing demand for plant-based foods with health-promoting properties, including plant proteins and plant lipids rich in unsaturated fatty acids. Plant-based foods produced from oilseeds (e.g., sunflower seeds, linseeds, rapeseeds, chia seeds and hemp seeds) are important sources of dietary fibre, nutrients providing proteins with essential amino acids and lipids, including the monounsaturated fatty acids (oleic acid, 18:1 *n*-9), the essential omega-3 (ω3) polyunsaturated fatty acids (*n*-3 PUFA, α-linolenic acid, ALA, 18:3 *n*-3) and the omega-6 (ω6) PUFA (*n*-6 PUFA, linoleic acid, 18:2 *n*-6), but also phytosterols and natural antioxidants such as tocopherols and phenolic compounds. These oilseeds are currently consumed in the form of whole seeds and oil extracted from the seeds. However, the natural lipid assemblies formed within these oilseeds that are indiscriminately called oil bodies (OBs), lipid droplets or oleosomes could be valorised for innovative food applications such as natural oil-in-water emulsions, as previously reported [1,2,3,4]. OBs have a core rich in triacylglycerols (TAGs: triesters of fatty acids and glycerol), covered by a hemi-membrane that corresponds to a monolayer of phospholipids and proteins (mainly oleosins and caleosins) [5,6]. This interfacial hemi-membrane is responsible for the high stability of OBs in aqueous medium. The use of OBs as a formulated emulsion may be of considerable utility in the food industry. Furthermore, OBs contain many valuable natural compounds, such as phospholipids, sterols and antioxidants (tocopherols and phenolics), that are involved in the oxidative stability of PUFA-rich OBs. The main advantages of using OBs (natural lipid droplets) as alternatives to oil (non-emulsified lipids) originate from their natural structure and composition that provide protection against oxidation [7]. 

Exploring the biodiversity of oilseeds with a specific focus on natural OBs and designing new green refinery processes for their soft extraction are therefore of primary interest to develop sustainable and healthy new ingredients for food applications.

Among oilseeds, traditional chia (*Salvia hispanica* L.) seeds and non-traditional camelina (*Camelina sativa* L.) seeds are of special interest in food applications [8,9]. These oilseeds have high nutritional and functional values associated with their abundant oil content and their chemical composition which is rich in essential PUFA, mainly ω3 PUFA, as well as proteins, dietary fibre and other bioactive compounds (phenolic compounds, tocopherols and plant sterols). These ω3 PUFA-rich seeds are therefore promising sources of plant nutrients for human consumption and are of special interest to increase the low ω3 dietary intake of the population as well as to improve the ω6/ω3 balance. 

Chia is an annual herbaceous plant of the Lamiaceae family, originally cultivated in tropical and subtropical mountainous regions. Nowadays, chia has adapted to greenhouse systems, allowing its seeding in different climates and all over the world, including Europe and France [10]. Chia seeds contain a high amount of lipids (30–33%), carbohydrates (26–41%), dietary fibre (18–30%), proteins (15–25%), vitamins, minerals and natural antioxidants [11,12,13]. Chia seeds are the richest plant source of ω3 PUFA known today with the ALA that accounts for about 60–65% of total chia seed fatty acids. Recent reviews have summarised the evidence on the beneficial clinical effects of chia seeds in humans [14,15]. In the European Union, the attraction of chia seeds in human diets and health has increased during the last decade due mainly to acceptation of chia for human consumption as a novel food ingredient [12,16,17]. Currently, chia seeds are consumed as ingredients in the form of whole seeds, seed flours (grounded seeds) and seed oil. Chia seeds are added to many foodstuffs including baked products, muesli, dairy drinks, fruit smoothies or salads.

Camelina is an annual sustainable oilseed crop within the Brassicaceae family, with emerging ongoing interests [18]. Today, camelina cultivation is low with production in North America, Russia and Europe. However, it is garnering renewed attention due to agronomic qualities and environmental attributes (e.g., adaptability to diverse environmental conditions, low requirements for water and nutrients and relatively strong resistance to insect pests and microbial diseases) [18,19,20]. Camelina is a climate-resilient oilseed crop that differs from other traditional oilseed crops. Among oilseeds containing interesting fatty acid profiles, camelina is identified as one of the main candidates to be used in the future European bioeconomy [21,22]. Camelina seeds contain a high amount of lipids (27–49%) and proteins (24–31%), as well as dietary fibre, carbohydrates, vitamins, minerals, and natural antioxidants [18,19]. Camelina oil contains from 50.8 to 66.6% PUFA with 31–40% ALA that contribute to the interest in its human nutrition and health benefits. The consumption of camelina oil provides health benefits, such as reductions in blood serum cholesterol levels and improvements in serum lipid profiles, as recently reviewed [19]. Currently, camelina oilseeds are mostly used for non-food applications and have limited food applications, with cold-pressed oil or seeds used as food ingredients [19]. Despite the interesting chemical composition of camelina seeds, few research studies have been performed to explore their potential in food applications. 

Over recent years, there has been a growing interest in the development of innovative green (organic toxic solvent free) refinery processes able to prepare natural OBs from oilseeds of nutritional interests (sunflower, rapeseed, linseed and hemp) for food applications. Integrated processes including the soaking of the seeds, grinding and centrifugation have been successfully used to recover OBs along with proteins by aqueous extraction from oilseeds [1,3,4,23,24]. Currently, chia and camelina seed processing consists mainly of mechanical pressing of whole oilseeds possibly followed by solvent (n-hexane) extraction of the remaining oil in the press cake. These two main conventional processing methods have been widely used for oil extraction and recovery [20]. Both chia and camelina seeds are mucilaginous seeds, meaning that they have the capacity to produce and expel high viscosity mucilage when in contact with water. This mucilage has been the subject of several studies dedicated to extraction processes, characterisation and valorisation as aningredient that has high potential as a natural thickener, stabiliser and emulsifier for food, cosmetic and pharmaceutical applications [25,26,27,28,29]. The efficient recovery of OBs from chia and camelina seeds requires the removal of mucilage before aqueous extraction, for example by using ultrasound [25].

The functional and sensory properties of foods including oil-in-water emulsions are affected by the size and surface properties of the lipid droplets. Furthermore, food products can have a neutral pH (e.g., plant beverages) or an acidic pH (e.g., fermented food products, yogurt, acidic cream and ice cream) that affects the surface properties of the lipid droplets and their physical stability (e.g., leading to aggregation or coalescence). In the perspective of a wide range of food applications, it is of interest to examine the surface properties and the microstructure of natural OBs recovered by aqueous extraction from oilseeds and to investigate their physical stability as a function of pH, as previously performed, for example, with hemp seed OBs [1,23]. 

In this work, the research hypothesis is that natural oil-in-water emulsions containing OBs recovered from oilseeds could be valorised for food applications as alternatives to the seeds or oils that are currently consumed. The objectives of this study, focused on chia and camelina oilseeds, were (i) to use a green refinery process able to recover aqueous extracts and OBs from the seeds, (ii) to examine the microstructure of chia seed and camelina seed OBs, (iii) to characterise the chemical composition of chia seed and camelina seed OBs, and (iv) to investigate with a multi-scale approach the physical stability of the natural oil-in-water emulsions containing OBs as a function of pH. This study was performed with a combination of chemical analysis (analysis of lipids including the fatty acids, molecular species of TAG, regiospecific distribution of fatty acids in TAG, plant sterols, tocopherols, phenolics and proteins), physico-chemical characterisations (particle size measurements and zeta-potential measurements) and microscopic observations using confocal laser scanning microscopy with pertinent fluorescent probes. Oxidative stability measurements of chia seed and camelina seed natural OBs were beyond the scope of this study and will be developed in the near future in a comparison with processed OBs.

## 2. Materials and Methods

### 2.1. Chia Seeds and Camelina Seeds

Chia (*Salvia hispanica* L.) seeds from organic agriculture were produced in Bolivia by Natural Andes (black chia seeds from agro-ecological agriculture) and provided by Biocoop Scarabée (35 Saint-Grégoire, France) once and in a sufficient amount for all the experiments performed in this study. 

Camelina (*Camelina sativa* L.) seeds were harvested from Camelina sativa cultivar “Celine” grown in open field at INRAE Versailles (France) during spring 2019 and maintained at 4 °C for long-term storage. Camelina seeds were provided once and in a sufficient amount for all the experiments performed in this study.

### 2.2. Sample Preparation

#### 2.2.1. Plant Seed Processing: Preparation of Seed Aqueous Extracts and Oil Bodies

**Figure 1:**
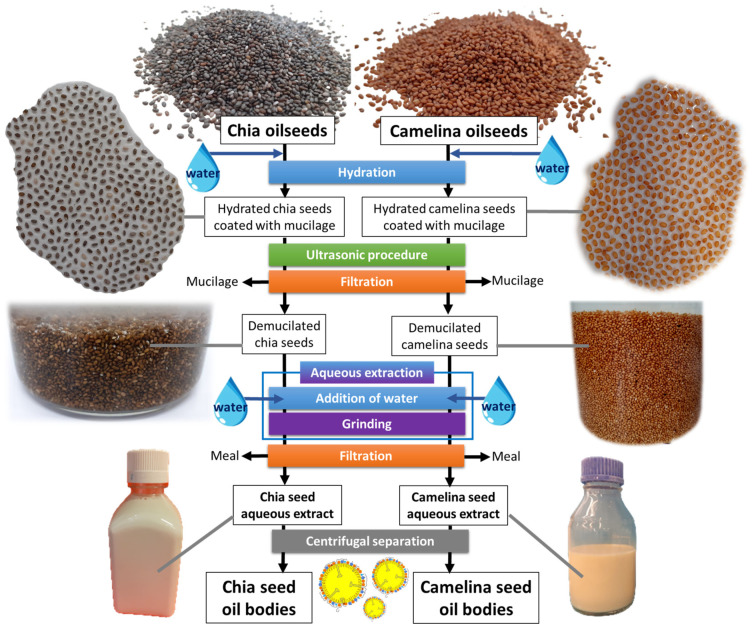
Schematic diagram of the integrated green refinery process used for the release of oil bodies after aqueous extraction from chia seeds or camelina seeds. Images show the chia and camelina seeds, hydrated chia and camelina seeds coated with mucilage, mucilage-free chia and camelina seeds obtained after ultrasonication and removal of the mucilage and the aqueous extracts.

The sequential refinery process employed in this study for the preparation of OBs from chia and camelina seeds involved the combination of environmentally friendly techniques according to the green approach since no organic solvent was used (Figure 1). The first step consisted of the hydration of the seeds and removal of the mucilage firmly attached to the seeds with a method adapted from [25]; the other steps were similar to the aqueous extraction process previously developed for hemp seeds [1]. 

Chia seeds or camelina seeds (batches of 200 g) were soaked in ultrapure water (1:5 *w/v*) and stirred in a mechanical stirrer for 12 h at 4 °C to allow their hydration. Upon hydration, chia seeds and camelina seeds were coated with a swollen transparent mucilage gel layer that remained firmly attached to their shell and separated the seeds (see pictures in Figure 1). The formation of mucilage required an ultrasonic procedure to separate it from the seeds. The technique of ultrasonication entails propagation of mechanical waves, generating cavitation microbubbles. The subsequent collapse of the bubbles is able to break the bonds between the seed coat and the mucilage. The hydrated seeds surrounded by mucilage were submitted to ultrasound operating at a frequency of 20 kHz and a power of 40 W for 20 min, with 30 s on and 10 s off using an ultrasonic processor (Misonix Sonicator S-4000, Qsonica, CT, USA) equipped with a microtip probe #418 (tip diameter 3.2 mm). During the ultrasonic procedure, the container was kept on ice to avoid an increase in temperature. The parameters of the ultrasonic procedure (duration, ultrasound power and temperature) were adapted to avoid damage to the seeds. The demucilated seeds were separated from the mucilage through filtration using a metallic filter with pores of 0.2 cm. The hydrated and demucilated chia and camelina seeds were collected for the next step of processing. 

An aqueous extraction including a mechanical treatment of grinding in water was applied to recover aqueous extracts from chia and camelina seeds containing the oil bodies (OBs), as previously developed for hemp seed OBs [1]. The hydrated and demucilated chia seeds or camelina seeds were ground in water for 3 min at a speed of 6000 rpm (Turbomix plus, Moulinex, France) to disrupt the cell walls and release the cellular material. The resulting slurry was then filtered through a layer of cheesecloth to remove the external part of the seeds (shells and solid residues) and cell wall components (composition confirmed by microscopic observations). The filtrates containing the OBs and proteins corresponded to the chia seed or camelina seed aqueous extracts. Sodium azide (NaN_3_, 0.01%) was added to the aqueous extracts to prevent the growth of bacteria. Chia and camelina seed aqueous extracts are natural oil-in-water emulsions that contain OBs and proteins. In order to isolate OBs, chia and camelina seed aqueous extracts were centrifuged at 4000× *g* for 30 min at 20 °C (Eppendorf^®^ 5810R centrifuge, Merck KGaA, Darmstadt, Germany). The layer of OB-rich cream at the top of the tubes was manually collected.

#### 2.2.2. Changes in pH

The OBs obtained by centrifugation were dispersed in water to reach the final concentration of 10% wt of lipids in the form of OBs. The pHs of the natural OB-based emulsions were adjusted in the range from pH 9 to pH 3 using 0.1 M HCl or NaOH solutions. Each emulsion was stored in 15 mL containers. To follow the physical stability of the emulsions as a function of time, the samples were stored at room temperature for 4 h and then stored at 4 °C for 2 days. Pictures of the containers were taken to characterise the physical stability of the OB-based emulsions as a function of pH.

### 2.3. Microstructure

Confocal laser scanning microscopy (CLSM) combined with DIC was used to examine the microstructures of chia and camelina seed aqueous extracts and OBs, as previously performed with hemp seed samples [1]. A microscope, NIKON A1R (NIKON, Champigny sur Marne, France), was used with a x60 oil immersion objective. The fluorescent Nile Red dye (100 µg/mL in propanediol; 5H-benzo-alpha-phenoxazine-5-one,9-diethylamino; Sigma Aldrich, St Louis, MO, USA) was used to stain the core of OBs rich in hydrophobic triacylglycerols (excitation wavelength = 560 nm). The fluorescent dye Fast Green FCF (10 mg/mL in water; Sigma-Aldrich, St. Louis, USA) was used to stain the proteins (excitation wavelength = 636 nm). The fluorescent dye Rh-DOPE (Lissamine rhodamine B sulfonyl dioleoylphosphatidylethanolamine; 1 mg/mL; Avanti Polar Lipids Inc., Birmingham, England) was used to visualise the monolayer of phospholipids surrounding chia and camelina seed OBs (excitation wavelength = 560 nm), as previously described for milk fat globules [26] and hemp seed OBs [1]. The samples were kept at room temperature for at least 20 min prior to microscopic observations.

### 2.4. Analysis of Proteins by Gel Electrophoresis

Sodium dodecyl sulphate polyacrylamide gel electrophoresis (SDS-PAGE) was used to determine the protein profiles in the chia and camelina seed aqueous extracts, and the interfacial protein composition of OBs obtained after centrifugation, as previously performed for hemp seed samples [1]. 

#### 2.4.1. Preparation of the Samples for Gel Electrophoresis

Washing of OBs was performed to remove exogenous proteins from the interface. Briefly, 5 g of OB-rich cream was added to 5 g of a solution containing 50% wt saccharose. Then, 10 mL of the treated OBs were deposited into 30 mL of a solution containing 5% wt saccharose in 45 mL plastic centrifuge tubes. Centrifugation of the tubes was performed at 4000× *g* for 30 min. The washed OBs recovered at the top of the tubes were used for SDS-PAGE analysis.

#### 2.4.2. Gel Electrophoresis

The protein compositions of the samples were characterised by SDS-PAGE with Mini-Protean TGX Precast Gels gradient 4–15% using the Mini-Protean Tetra Cell system (Bio-Rad Life Science, France). All reagents were from Bio-Rad Life Science. The samples were diluted with 2 × Laemmli denaturing sample buffer in reducing conditions with 2-mercaptoethanol 5% (2-ME; a reducing agent that breaks down the disulphide bonds). The protein compositions of chia and camelina seed aqueous extracts were also examined in non-reducing conditions, by using the sample buffer in the absence of 2-ME. The migration buffer was composed as follows: 25 mM Tris, 192 mM glycine and 0.1% SDS, according to the protocol of [27]. The samples were heated at 100 °C for 5 min. Each protein sample was then loaded onto a sample well of the gradient gel. Molecular weight (MW) protein markers with MWs of 10 to 250 kDa (Precision Plus Protein Standards, All Blue, Bio-Rad Life Science, France) and with MWs from 14.4 to 116 kDa (unstained Molecular weight marker, Euromedex, Souffelweyersheim, France) were used for MW calibration. The migration was performed at 200 V for 45 min. The gels were stained with Coomassie Brilliant Blue G-250 staining solution for 2 h under gentle agitation, according to [28]. Then, each gel was rinsed with distilled water. The gels were then scanned on a flatbed scanner (Image Scanner iii, GE Healthcare Europe, Velizy- Villacoublay, France).

### 2.5. Particle Size Measurements

A laser diffraction analyser (Horiba LA-960V2, Retsch Technology, Haan, Germany) was used to determine the size distributions of chia and camelina seed OBs. The refractive indexes used were 1.47 for the OBs and 1.33 for the continuous phase (water), as previously reported [1]. The samples were characterised in two conditions: (i) in water and (ii) after 10-fold dilution in sodium dodecyl sulphate (SDS 1% wt) to avoid flocculation between OBs and to determine the size distribution of individual OBs. The samples were added to ultrapure water for the particle size measurements. The measurements of each sample were performed in triplicate at room temperature. At least five independent samples of OBs were prepared for each kind of seed. The mean diameters were calculated with at least *n* = 15 values.

### 2.6. Chemical Analysis

#### 2.6.1. Total Lipids: Extraction and Quantification 

The total lipid content of the whole chia and camelina seeds was determined in triplicate by Soxhlet extraction with petroleum ether as a solvent. 

For the extraction of total lipids from the creams concentrated with chia or camelina seed OBs, the cold extraction procedure developed by Folch and collaborators [29] was used. Total lipid extractions were performed in triplicate, from three independent OB-rich creams (*n* = 9 lipid extracts for each kind of seed). These lipid extracts were used for chemical analyses, as detailed in the following paragraphs.

#### 2.6.2. Fatty Acid Profiles of Chia and Camelina Seed OBs

The compositions in fatty acids of chia and camelina seed OBs were determined by gas chromatography (GC). The fatty acid methyl esters (FAMEs) were prepared by methylation using a cold methanolic solution of potassium hydroxide, as previously described for hemp seed OBs [1]. FAMEs were analysed by a gas chromatograph (Focus GC, Thermo Electron Corporation, Waltham, MA, USA) equipped with a split injector (ratio of 1/20), a capillary column (CPCil 88 Varian) (50 m × 0.25 mm with a 0.2-μm thick film; Chrompack, Middelburg, The Netherlands) and helium as the carrier gas (1 mL/min). FAMEs were analysed using a flame ionization detector and the ChromCard Data System (Thermo Electron Corporation, Massachusetts, USA). The FAMEs were identified using a mixture of methyl esters as external standards (Mixture ME 100, Larodan, Sweden). The GC analysis was performed in duplicate for each total lipid extract from chia and camelina seed OBs (*n* = 9 lipid extracts, *n* = 2 GC analysis per lipid extract; *n* = 18 fatty acid compositions for each kind of seed).

#### 2.6.3. Analysis of Triacylglycerols (TAGs)

##### Recovering of TAGs 

Chia and camelina seed oils (0.12 g of lipid extract; *n* = 9 lipid extracts for each kind of seed) were used. Silica gel column chromatography was performed on the oils using Sep-Pak silica (1 g, Waters Corp. Milford, MA, USA), preequilibrated by 10 mL of hexane:diethyl ether (87:13 *v*/*v*). TAGs were recovered by eluting the column with 10 mL hexane:diethyl ether (87:13 *v*/*v*). The solvents were removed under reduced pressure. The TAGs recovered were then used for the TAG and FA distribution analyses.

##### Analysis of the Molecular Species of TAGs

Analysis of the molecular species of TAGs in chia and camelina seed OBs was conducted by the official assay [30], for the separation, identification and quantitative determination of individual TAGs in edible fats and oils using HPLC. We used an HPLC-ELSD (Agilent 1200, Agilent Technologies, Santa Clara, CA, USA) equipped with a C18 column (150 mm × 4.6 mm, 5 µm, Varian, Palo Alto, CA, USA). The oils were prepared in hexane at 2 mg/mL. The oils were then analysed. The mobile phase consisted of acetonitrile and isopropanol (flow rate 0.8 mL/min). The initial mobile phase was held for 40 min at 60% acetonitrile, then changed linearly (40–80 min) to 55%, returned to 60% (80–85 min) and finally maintained for 5 min (85–90 min) at 60%. The ELSD temperature was set at 55 °C (gain value of 8; gas flow rate of 1.5 mL/min). TAGs were separated based on the equivalent carbon number (ECN), which was calculated with the formula: ECN = TCN − (2 DB), where TCN is the total carbon number and DB is the number of double bonds in the FA in each TAG. TAGs were identified by comparing their retention times with standard reference compounds. Relative content was reported as a percentage. From chia seed lipid extracts (*n* = 9) and camelina seed lipid extracts (*n* = 9), the molecular species of TAGs were determined in duplicate (two injections in the HPLC); the results presented correspond to the mean of *n* = 18 chromatograms for each kind of seed.

##### Analysis of the Fatty Acid Distribution in TAGs: Production of *sn*-2 MAG

For the positional distribution of fatty acids in TAGs, the Joint JOCS/AOCS Official Method Ch 3a-19 [31], that uses immobilised *Candida antarctica* lipase, was chosen. This is a simple method that simultaneously analyses the regiodistributions of fatty acids including PUFA [32,33]. In brief, 0.04 g of TAGs, 1.0 mL of ethanol and 40 mg of immobilised *Candida antarctica* lipase (Lipase B *Candida antarctica* immobilised on Immobead 150, recombinant from yeast > 2000 U/g; Sigma) was mixed and shaken for 3 h at 30 °C. The reaction mixtures (approximate glyceride composition: 60% FAEE, 30% *sn*-2 MAG and 5% DAG; [34]) were separated from the immobilised lipase. Ethanol was then removed under reduced pressure. The mixture was immediately brought to Sep-pak silica (0.69 g), pre-equilibrated by 10 mL of hexane:diethyl ether (8:2, *v*/*v*). After rinsing the column with 30 mL of hexane:diethyl ether (8:2, *v/v*), the *sn*-2 monoacylglycerol (*sn*-2 MAG) fraction was recovered by eluting 10 mL of diethyl ether. For chia seed lipid extracts (*n* = 9) and camelina seed lipid extracts (*n* = 9), the TAG hydrolysis was performed in triplicate; the results presented correspond to the mean of *n* = 27 samples for each kind of seed.

##### Analysis of Fatty Acid Composition by Gas Chromatography

TAGs and *sn*-2 MAG fractions were methylated, in duplicate, by the potassium hydroxide–methanol method described in [35] with slight modifications. Briefly, the acylglycerols were dissolved in 2 mL iso-octan. A volume of 0.2 mL of 2 mol/L potassium hydroxide–methanol was added to the sample and vortexed for 1 min. The solution was centrifuged at 2500× *g* rpm for 5 min, and the resulting upper layer was recovered. The FA composition was analysed by a 7890B (AGILENT) equipped with a CPSIL 88 capillary column (100 m × 0.25 mm, 0.20 μm; Agilent). The analytical program was 60 °C (2 min)–30 °C/min; 172 °C (5 min)–1 °C/min; 210 °C (22 min). The hydrogen pressure was 150 kPa, the injection ratio was 1/25, and the temperatures of the injector and FID detector were set at 250 °C. Each sample was analysed in duplicate (*n* = 2 injection in GC per sample). 

#### 2.6.4. Polar Lipid Analysis of Chia and Camelina Seed OBs

The total phospholipids recovered in chia and camelina seed OB samples were quantified using HPLC combined with an evaporative light scattering detector (ELSD), as previously described [1,36]. Identifications of the individual phospholipid classes were carried out by comparison with the retention times of pure standards. Quantification of individual phospholipids was performed using calibration curves. From chia seed lipid extracts (*n* = 9) and camelina seed lipid extracts (*n* = 9), the quantification of phospholipids was performed in triplicate; means were calculated with *n* = 27 for each kind of seed.

#### 2.6.5. Quantification of Sterols

The identification and quantification of total sterols were performed using gas chromatography with flame ionization (GC-FID) analysis according to [37], as previously described [1].. From chia seed lipid extracts (*n* = 9) and camelina seed lipid extracts (*n* = 9), the quantification of sterols was performed in duplicate; means were calculated with *n* = 18 for each kind of seed.

#### 2.6.6. Quantification of Tocopherols

The quantification of total and individual tocopherols was performed by HPLC on lipid extracts as previously described [1]. From chia seed lipid extracts (*n* = 9) and camelina seed lipid extracts (*n* = 9), the quantification of tocopherols was performed in triplicate; means were calculated with *n* = 27 for each kind of seeds.

#### 2.6.7. Quantification of Total and Individual Phenolic Compounds

The aqueous extracts of camelina and chia seeds were delipidated by homogenization with hexane (50/50 *v/v*). A centrifugation was performed at 10,000× *g* for 5 min (Thermo scientific Sorvall LYNX 6000 centrifuge) and the upper hexane phase containing the lipids was eliminated. The pHs of the delipidated samples were adjusted to a pH of 3 with a solution of malic acid 400 g/L, and then frozen and freeze-dried. The determination of the phenolic profile and the analysis of condensed tannins were performed by phloroglucinolysis followed by HPLC analysis, according to a method adapted from [38]. Briefly, 0.8 mL of methanol containing phloroglucinol (75 g/L) and ascorbic acid (15 g/L) was added to 50 mg of camelina and 100 mg of chia freeze-dried powders. Then, 0.4 mL of HCl (0.3 N in methanol) was added and the solutions were incubated at 50 °C for 30 min. Samples were chilled on ice for 5 min and the reactions were stopped by adding 1.2 mL of 0.2 M aqueous sodium acetate. The solutions were filtered on Vivaspin^®^ to eliminate proteins, then with PTFE 0.45 µm before HPLC analysis. The solvents used were (A) acidified pure water (0.1% formic acid) and (B) acidified acetonitrile (0.1% formic acid). The flow rate was 0.2 mL/min and the gradient was as follows: initial, 3% B; 0–3 min, 7% B, linear; 3–21 min, 13% B, linear; 21–27 min, 13% B, linear; 27–41 min, 20% B, linear; 41–51 min, 45% B, linear; followed by washing and reconditioning of the column. Identification of the phenolic compounds was performed by using the respective standard molecules (Sigma-Aldrich). Quantitation was performed by integrating peaks on UV−visible chromatograms at 280 nm for (−) epicatechin and at 320 nm for caffeic acic and sinapin by using the calibration curves of the respective standard molecules, and at 350 nm for rutin by using the calibration curves of equivalent hyperoside (hyperoside 1027S, Extrasynthese, France). The analysis of phenolics was performed from a single aqueous extract for each kind of seed. 

### 2.7. Zeta Potential Measurements

For zeta-potential (ζ-potential) experiments as a function of pH, dispersions of chia and camelina seed OBs were diluted in water (100 µL OB dispersion in 10 mL water) and the pHs of the dispersions were adjusted in the range of pH 9 to pH 3 using NaOH or HCl, as previously reported for hemp seed OBs [1]. About 1 mL of the diluted OB dispersion was placed into a cuvette. The cuvettes were placed into the chamber of a Zetasizer Nano ZS (Malvern, Germany) and equilibrated at 20 °C for 5 min before measurements. The zeta potential was calculated from the electrophoretic mobility of the OBs according to the Smoluchowski approximation and Henry’s law. The measurements were run five times at 20 °C on at least three independent and freshly prepared samples (means calculated with *n* = 15 values per pH). 

### 2.8. Statistical Analysis

Analyses of variance (ANOVA) was performed using the General Linear Model procedure of Statgraphics Plus version 5 (Statistical Graphics Corp., Englewood Cliffs, NJ, USA). Differences of *p* < 0.05 were considered significant. 

## 3. Results and Discussion

### 3.1. Microstructure of Chia and Camelina Seed Aqueous Extracts and OBs

**Figure 2:**
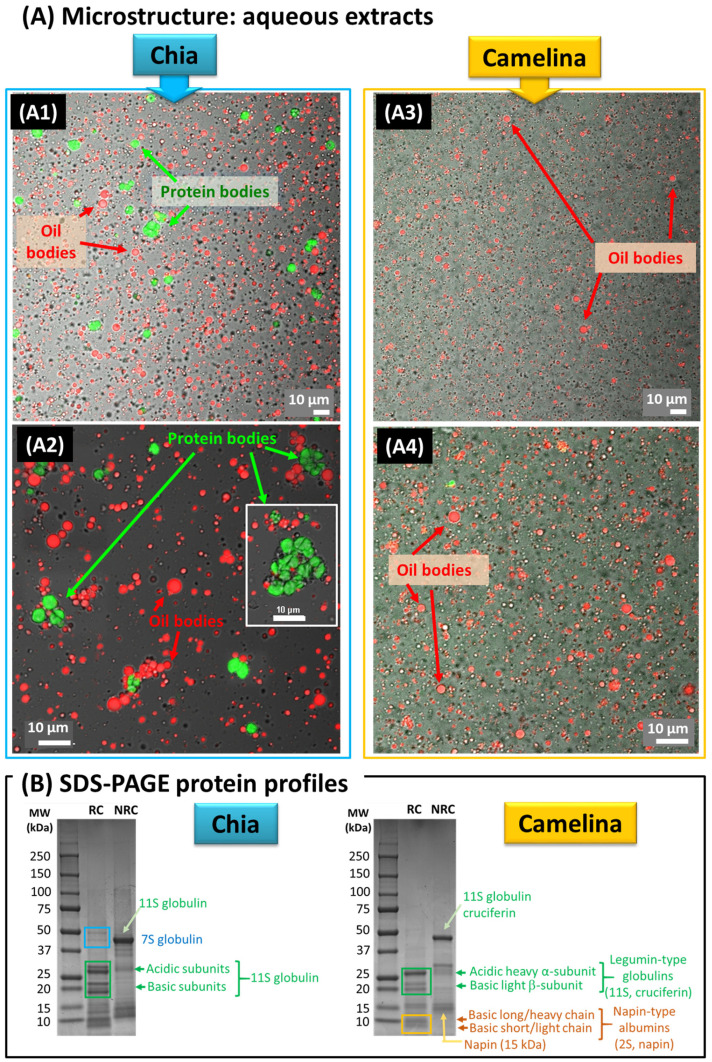
Microstructure of the aqueous extracts obtained from chia and camelina seeds. (**A**) Confocal laser scanning microscopy (CLSM) combined with DIC images of the chia seed (left; (**A1**,**A2**)) and camelina seed (right; (**A3**,**A4**)) aqueous extracts showing the dispersion of oil bodies and proteins (OBs: red colour, Nile Red fluorescent dye; protein bodies: green colour, Fast Green FCF fluorescent dye); (**B**) SDS-PAGE protein profiles of the chia seed and camelina seed aqueous extracts. Abbreviations: MW: molecular weight; NR: non-reducing conditions; RC: reducing conditions.

**Figure 3:**
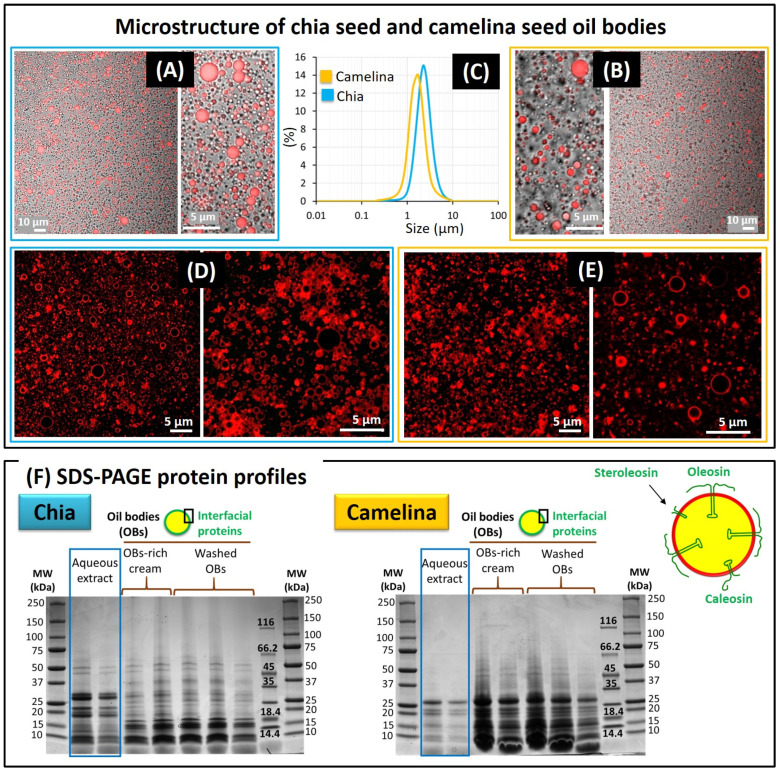
Microstructure of chia and camelina seed oil bodies (OBs) and identification of their interfacial proteins. (**A**,**B**) Confocal laser scanning microscopy (CLSM) combined with DIC images showing chia (**A**) and camelina (**B**) seed OBs (red colour: triacylglycerol core labelled with Nile Red fluorescent dye). (**C**) Size distribution of the chia and camelina seed OBs measured by laser light scattering; (**D**,**E**) CLSM images showing the monolayer of phospholipids around chia (**D**) and camelina (**E**) seed OBs (red colour: rhodamine-DOPE fluorescent dye); (**F**) SDS-PAGE protein profiles of chia and camelina seed aqueous extracts and OBs in the cream recovered after centrifugation and after washing (reducing conditions); (right) schematic representation of a OB with specific membrane proteins as indicated. Abbreviations: MW: molecular weight.

Figure 2 and Figure 3 show the microstructure of the chia seed and camelina seed aqueous extracts and OBs that were recovered by the green refinery process.

#### 3.1.1. Aqueous Extracts Obtained from Chia and Camelina Seeds

CLSM images combined with DIC showed the microstructure of chia seed (Figure 2A1,A2) and camelina seed (Figure 2A3,A4) aqueous extracts. The mechanical shear applied upon grinding of the hydrated and mucilage-free seeds during the aqueous extraction induced the release of spherical lipid droplets corresponding to the OBs (labelling of the TAG core in the CLSM images) and their dispersion in aqueous media. Chia and camelina seed aqueous extracts were therefore natural oil-in-water emulsions in which the oil droplets are the natural OBs of the seeds. 

At the pH of the aqueous extraction in water, i.e., pH of 6.4, the OBs formed aggregates (see impact of pH on the physical stability of OBs at the end of this paper). In the chia seed aqueous extracts, OBs coexisted with large protein bodies that correspond to chia seed storage proteins (Figure 2A1,A2). The presence of large protein bodies was reported for hemp seed aqueous extracts [1]. Such large protein bodies were not observed in the camelina seed aqueous extracts (Figure 2A3,A4).

The proteins present in the chia and camelina seed aqueous extracts were identified by SDS-PAGE (Figure 2B). 

Concerning chia seed aqueous extracts, the SDS-PAGE performed under reducing conditions showed the presence of four intense bands between 18 and 35 kDa, corresponding to the acidic (around 30 kDa) and basic (around 20 kDa) sub-units of the 11S globulin proteins. The two intense bands between 40 and 50 kDa were attributed to 7S globulins. The main chia seed storage proteins identified in the aqueous extracts were globulins. This is in agreement with their main proportion in chia seeds since globulins account for more than 50% wt of chia proteins, while other proteins such as albumins, prolamins and glutelins are present in chia seeds in low amounts [39]. In non-reducing conditions, the intense band around 48 kDa was interpreted as monomers of the 11S globulin proteins formed by the subunits identified in reducing conditions. A comparison between reducing and non-reducing conditions indicated that the major chia seed storage proteins, the globulins, contained disulphide bonds in their structure. The 11S globulin proteins have a typical hexameric conformation with a MW of around 300–400 kDa that was not revealed in the SDS-PAGE conditions used in this study. These high MW hexameric assemblies correspond to the protein bodies observed in the CLSM images (Figure 2A). 

Concerning the camelina seed aqueous extracts under reducing conditions, the SDS-PAGE revealed major bands at 15–25 kDa and 25–35 kDa that corresponded to the acidic heavy α-subunit (about 30 kDa) and the basic light β-subunit (about 20 kDa) of the legumin-type globulins (11S or cruciferin). The bands below 15 kDa were attributed to the napin-type albumins that contain two basic subunits, i.e., a long-chain subunit (about 10 kDa) and a short-chain subunit (about 4.5 kDa). This is in agreement with studies on the protein composition of camelina seeds and meals [40,41]. Under non-reducing conditions, the main band below 50 kDa was attributed to the 11S globulin cruciferin, formed by the acidic and basic subunits linked with one disulphide bond, while the band at 15 kDa corresponded to napin composed of the subunits linked with 1:1 disulphide linkage, in agreement with the literature [40,42]. Indeed, two types of seed storage proteins are abundant in Brassicaceae oilseeds including the camelina oilseeds: legumin-type globulins (11S or cruciferin) and napin-type albumins (2S or napin), which account for 60% and 20% of total proteins in the mature seeds, respectively [42].

#### 3.1.2. Oil Bodies Recovered from the Chia and Camelina Seed Aqueous Extracts

The centrifugation step involved in the green refinery process (Figure 1) allowed for the concentration of OBs in an OB-rich cream (fat content: 35–45% wt). Microscopy images show the chia and camelina seed OBs after labelling of their TAG core (Figure 3A,B). CLSM images of chia samples revealed the absence of protein bodies indicating that centrifugation induced the sedimentation of protein bodies in agreement with their high density, as previously reported for the sedimentation of edestin protein bodies in hemp seed aqueous extracts [1]. The size distributions of chia seed OBs and camelina seed OBs recovered in the OB-enriched fractions obtained after centrifugation are presented in Figure 3C. The mean diameters of OBs were determined by laser light scattering. The size distributions were monomodal. Chia seed OBs exhibited a mean diameter of 2.3 ± 0.1 µm and a size distribution ranging from 0.4 to 10 µm. The mean diameter of camelina seed OBs was 1.6 ± 0.2 µm, with a size distribution ranging from 0.2 to 10 µm. The mean diameter of camelina seed OBs was significantly (*p* < 0.005) smaller than the mean diameter of chia seed OBs (*n* = 5 independent extractions from chia and camelina seeds). These mean diameters of OBs in the range of 2 µm are in accordance with reports on natural OBs from other origins. The diameters of sesame and peanut Obs were reported to be 2.4 ± 0.4 μm and 2.3 ± 0.3 μm, respectively, with sizes ranging from 1 to 10 µm [43]. Using aqueous extraction, the mean diameter of hemp seed OBs was 2.3 ± 0.1 μm [1] and the mean diameter of sunflower OBs was 2.6 ± 0.1 μm [44]. 

**Figure 4:**
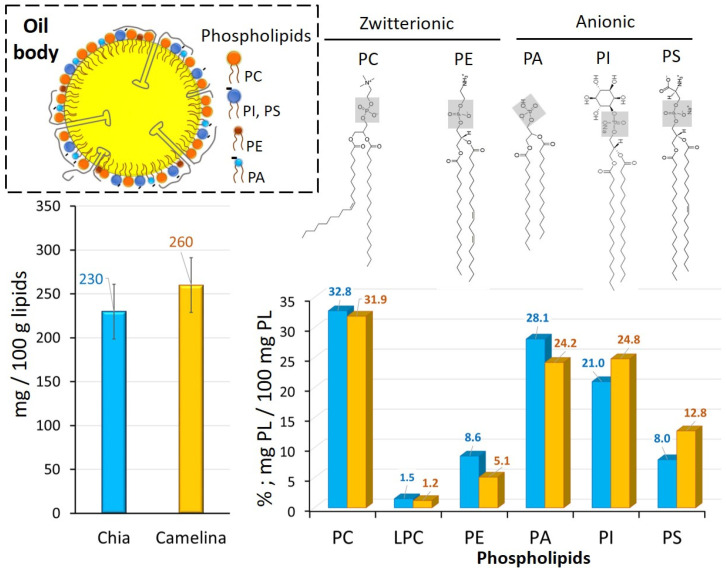
Phospholipid composition of chia and camelina seed oil bodies (OBs). Total phospholipid amounts and relative percentage of the main individual phospholipids. Schematic representation of an oil body enveloped by the monolayer of phospholipids (top left). The chemical structures of the main phospholipids are indicated in the figure (top right). Abbreviations: PC: phosphatidylcholine, LPC: lyso-phosphatidylcholine, PE: phosphatidylethanolamine, PA: phosphatidic acid, PI: phosphatidylinositol, PS: phosphatidylserine.

CLSM images with the Rh-DOPE fluorescent dye showed the phospholipids located at the TAG/water interface in the hemi-membrane surrounding the chia and camelina seed OBs (Figure 3D,E). At the microscopic scale level, the phospholipids appeared to be organised as a homogeneous layer, as previously reported for hemp seed OBs [1,23]. The presence of this monolayer of phospholipids at the surface of the OBs indicated that the OBs extracted from the seeds were not altered during the processing and corresponded to natural OBs. Indeed, intense shearing gradients during the mechanical treatment and homogenisation of aqueous extracts containing OBs lead to the adsorption at the TAG/water interface of proteins previously located in the aqueous phase, as formerly reported for hemp seed OBs [1]. The phospholipid composition of chia and camelina OBs is provided in Figure 4.

SDS-PAGE allowed the identification of the proteins contained in the aqueous extracts and of those specifically localised at the surface of the chia and camelina seed OBs (Figure 3F). In the chia seed OB samples, SDS-PAGE showed intense bands with MWs in the range of 15–18 kDa and a band at 25 kDa; these were interpreted as oleosins. The band at 35 kDa was interpreted as the caleosins and the bands at 35–60 kDa were attributed to steroleosins. For the camelina seed OB samples, similar membrane-specific proteins, i.e., oleosins, caleosins and steroleosins, were also revealed by SDS-PAGE. These proteins are the major specialised integral membrane proteins identified at the surface of OBs [3,5]. They are therefore located at the surface of the natural OBs obtained by aqueous extraction together with the phospholipids to form the hemi-membrane. A schematic representation of OBs with specific membrane proteins is presented in Figure 3F.

### 3.2. Composition

#### 3.2.1. Lipid Content of the Chia and Camelina Seeds

The lipid content of the chia seeds used in this study was 35.3 ± 0.2 g/100 g, which was in accordance with the literature [9,45,46]. The camelina seeds contained 42.1 ± 1.6 g/100 g of lipids, also in agreement with previous reports [18,19]. Both the chia and camelina seeds are rich in oil and are therefore suitable oilseeds for the preparation of foods such as oil-in-water emulsions. 

#### 3.2.2. Phospholipid Composition of OBs

The total amount of phospholipids and the relative amount of individual phospholipids were determined in the chia and camelina seed OB samples (Figure 4). The amount of phospholipids in chia seed OBs was 0.23 ± 0.3 g/100 g of oil. Four main phospholipid classes were identified and were accounted for in the following order: 32.8% phosphatidylcholine (PC), 28.1% phosphatidic acid (PA), 21% phosphatidylinositol (PI), 8.6 % phosphatidylethanolamine (PE), 8% phosphatidylserine (PS) and 1.5 % lyso-PC. There is currently little information in the literature on chia seed phospholipid composition, but these results are in agreement with [47]. The same classes were also found in chia seed oil by [48], with the exception of lyso-PC. In camelina seed OBs, the amount of phospholipids was 0.26 ± 0.4 g/100g of oil. The individual phospholipids corresponded to 31.9% PC, 24.2% PA, 24.8% PI, 12.8% PS, 5.1% PE and 1.2% LPC. These results are in accordance with [49]. Similar amounts of total phospholipids and individual classes of phospholipids were found for hemp seed OBs [1]. The anionic phospholipids such as PA, PI and PS contain negative charges that contribute to the electrostatic repulsions between OBs dispersed in an aqueous phase. These anionic phospholipids accounted for 57.1% and 61.8% of total phospholipids in the chia and camelina seed OBs, respectively. Phospholipids are a minor lipid fraction of OBs located at the TAG/water interface that play an important functional role in association with membrane proteins and their physical stability, as reported for OBs from various origins [1,23]. 

#### 3.2.3. Fatty Acid and Triacylglycerol Composition of OBs

The lipid compositions of the seeds and the oils extracted from chia and camelina seeds have been investigated [8,9,20,50]. However, there are no data in the literature concerning the lipid composition of the chia and camelina seed OBs. In this study, we focussed on the fatty acid composition and on the TAG composition, including the molecular species of TAG and the regiospecific distribution of the fatty acids on the glycerol backbone.

##### Fatty Acid Composition

**Figure 5:**
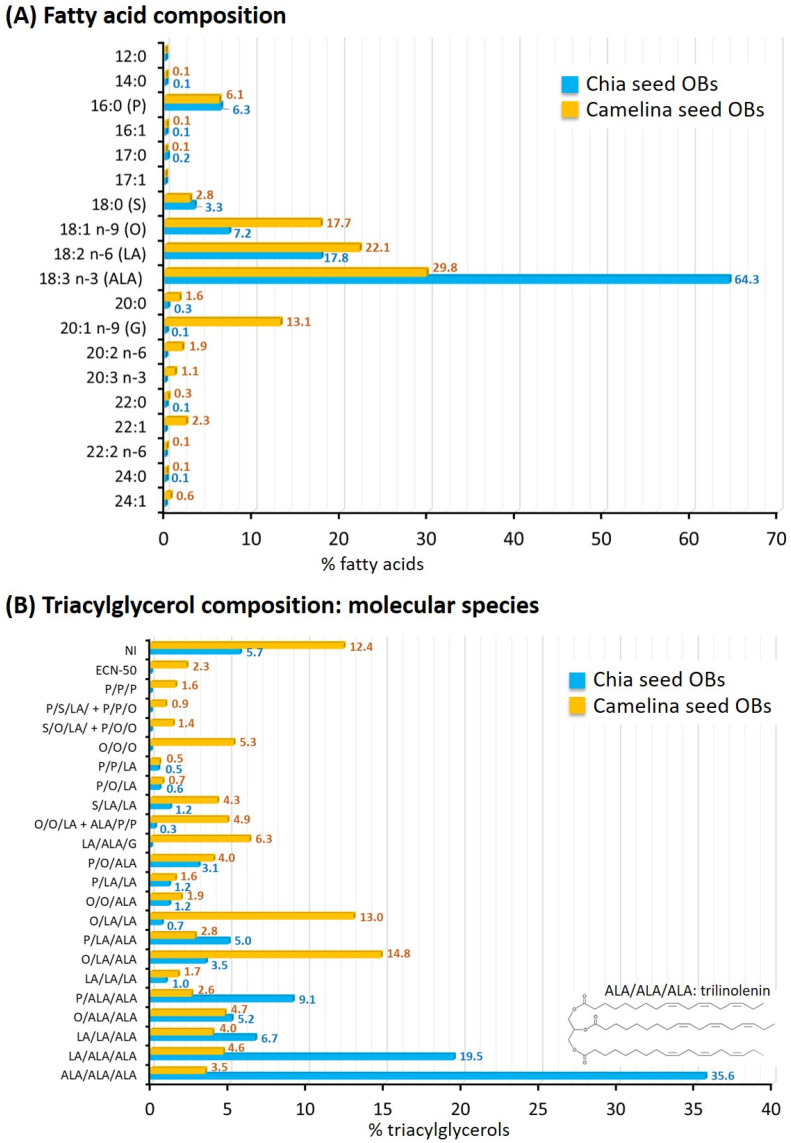
Lipid composition of chia and camelina seed oil bodies (OBs). **(A)** Fatty acid composition (g/100 g total fatty acids), (**B**) composition of triacylglycerol (TAG) molecular species (g/100 g total TAG). Abbreviations: ALA = α-linolenic acid, LA = linoleic acid, O = oleic acid, P = palmitic acid, S = stearic acid and G = gondoic acid; symbols O/LA/ALA, LA/ALA/ALA and others indicate the three fatty acids present in TAG but do not represent their regiospecific *sn*-position. Values are reported as means ± SD of three independent extractions and 3 analyses per extraction (*n* = 9).

The fatty acid compositions of chia seed and camelina seed OBs are presented Figure 5A. Analysis of the chromatograms revealed five to six main fatty acid species. In both chia seed and camelina seed OBs, α-linolenic acid (ALA; 18:3 *n*-3) dominated among the fatty acids, accounting for 64.3 ± 0.2% and 29.8 ± 0.1% of total fatty acids, respectively. Camelina and chia seed OBs therefore represent interesting plant-based sources of ALA in the human diet. However, according to the biodiversity of oilseeds, the amount of ALA was 2.2-fold higher in chia seed OBs compared to camelina seed OBs. The other main fatty acids found in chia and camelina seed OBs were linoleic acid (LA; 18:2 *n*-6), oleic acid (O; 18:1 *n*-9), palmitic acid (P; 16:0) and stearic acid (S; 18:0) in decreasing amounts. The camelina seed OBs contained 13.1 ± 0.1% of eicosenoic acid (20:1 *n*-9; also called gondoic acid or gadoleic acid; G), as previously reported for camelina seed oil [8,51,52], while chia seed OBs contained 0.1% of this fatty acid, in agreement with the literature [45]. In the camelina seed OBs, the concentration of erucic acid (22:1 *n*-9) was 2.34%, in agreement with data reported for camelina seed oil [53]. Camelina seed oil has a lower content of erucic acid (1.6–4.2%) compared with other oils of cruciferous species [54]. 

The total PUFA accounted for 82.1 ± 0.2% in chia seed OBs and 55.0 ± 0.1% in camelina seed OBs. The monounsaturated fatty acids represented 7.5 ± 0.1% and 33.9 ± 0.1% in chia and camelina seed OBs, respectively. The saturated fatty acids represented 10.4 ± 0.1% and 11.1 ± 0.1% of total FA in chia and camelina seed OBs, respectively. The ω6/ω3 PUFA ratio was 0.28 for chia seed OBs and 0.78 for camelina seed OBs. This was in agreement with the literature, since authors have reported a markedly low ratio of around 0.3–0.35 for chia seed oil [9,47,50,55] and ratios ranging from 0.4 to 1.6 for camelina seed oil. These low ω6/ω3 PUFA ratios are due to the high amount of ALA in chia and camelina seed OBs. Chia and camelina seed OBs have the potential to reduce the ω6/ω3 PUFA ratio in the human diet.

The fatty acid composition of chia seed OBs was in agreement with previous studies dedicated to chia seed oil [9,46,55,56]. The fatty acid composition of camelina seed OBs was also in agreement with data reported for camelina seed oil [19]. In conclusion, the aqueous extraction of OBs from the seeds did not affect the fatty acid composition when compared to the oil obtained by pressing of the seeds.

##### Triacylglycerols: Molecular Species and Distribution of the Fatty Acids on the Glycerol Backbone

Triacylglycerols (TAGs) accounted for approximately 95–97% of the chia and camelina seed lipids extracted from the OBs. Figure 5B shows the molecular species of TAGs in chia and camelina seed OBs. 

In chia seed OBs, six TAG species accounted for more than 4%. We found that the trilinolenin (ALA/ALA/ALA; ALA = 18:3 *n*-3) was the main TAG, which is in accordance with the FA profile of chia seed OBs and the high amount of ALA (64.3 ± 0.2% of total FA; Figure 5A). Our results are in agreement with the identification of trilinolenin as the major TAG in chia seed oil [9,46,50]. The other main TAGs were linoleoyl-linolenoyl-linolenoylglycerol (LA/ALA/ALA), palmitoyl-linolenoyl-linolenoylglycerol (P/ALA/ALA), linoleoyl-linoleoyl-linolenoylglycerol (LA/LA/ALA), oleoyl-linolenoyl-linolenoylglycerol (O/ALA/ALA) and palmitoyl-linoleoyl-linolenoylglycerol (P/LA/ALA). These TAG molecular species contained the main FA found in chia seed OBs. Linolenic acid (ALA) was present in most of the TAGs found in chia seed OBs. This TAG profile was in agreement with recent TAG compositions determined for chia seed oil [46,50].

In camelina seed OBs, nine TAG accounted for more than 4% (Figure 5B). The main TAGs were oleoyl-linoleoyl-linolenoylglycerol (O/LA/ALA; 14.8%) and oleoyl-linoleoyl-linoleoylglycerol (O/LA/LA; 13%). Then, the TAG containing the three most abundant fatty acids (oleic acid, linoleic acid and linolenic acid) accounted for 22.1% of the total TAG (ALA/ALA/ALA, LA/ALA/ALA, O/ALA/ALA, LA/LA/ALA and OOO). The TAG linoleoyl-linolenoyl-gondoylglycerol (LA/ALA/G) accounted for 6.3% of the TAG as a result of the abundance of gondoic acid (G; 20:1 *n*-9; 13.1 ± 0.1% of total FA; Figure 5A) among the camelina seed fatty acids. 

**Figure 6:**
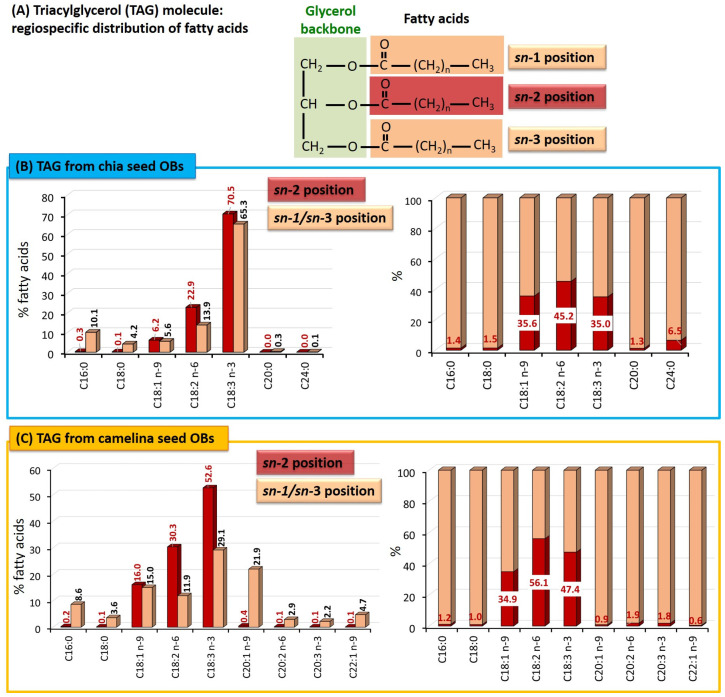
Regiospecific distribution of the fatty acids in the triacylglycerols (TAG) from chia and camelina seed oil bodies (OBs). (**A**) Schematic representation of a TAG molecule showing the central *sn*-2 position and the external *sn*-1 and *sn*-3 positions. (**B**) TAGs from chia seed OBs; left: relative proportion of the fatty acids in the *sn*-2 position and in the *sn*-1/3 positions (% total fatty acids in the same position); right: relative distribution of each fatty acid in the *sn*-2 and *sn*-1/3 positions; (**C**) TAGs from camelina seed OBs; left: relative proportion of the fatty acids in the *sn*-2 position and in the *sn*-1/3 positions (% total fatty acids in the same position); right: relative distribution of each fatty acid in the *sn*-2 and *sn*-1/3 positions.

In this study, the regiodistribution of the fatty acids in the TAG molecules contained in chia and camelina seed OBs was examined. The position of the fatty acids esterified on the glycerol moiety in TAG molecules (stereospecific numbering *sn*; *sn*-1, *sn*-2 and *sn*-3; Figure 6A) produces a characteristic stereospecificity corresponding to the internal structure of TAG molecules. This stereospecificity defines the physical properties of the oils and influences their digestion in the gastro-intestinal tract, absorption, metabolism and uptake into tissues. Importantly, several studies over the last three decades have shown that the fatty acids located in the *sn*-2 position (central position of the TAGs; *sn*-2 monoacylglycerol formed upon digestion [57,58]) promote a more efficient absorption of the lipids by the intestinal epithelium, and have different metabolic fates than the fatty acids in the *sn*-1 and *sn*-3 positions [57,59,60,61].

Figure 6 shows the fatty acid composition at the *sn*-2 and *sn*-1/3 positions and the preferential distribution of each main fatty acid between the internal *sn*-2 position or the external *sn*-1/3 positions that have been determined for the TAGs contained in the chia seed OBs (Figure 6B) and the camelina seed OBs (Figure 6C). 

In chia seed OBs (Figure 6B), the fatty acids esterified at the *sn*-2 position of the TAGs were mainly α-linolenic acid (70.5 %; 18:3 *n*-3, ALA), linoleic acid (22.9 %; 18:2 *n*-6, LA) and oleic acid (6.2 %; 18:1 *n*-9, O); representing 99.5% of the total fatty acids in the *sn*-2 position. The ω6-LA/ω3-ALA ratio in the *sn*-2 position of chia seed TAGs was 0.3, which is close to the ω6/ω3 PUFA ratio of 0.28 found for chia seed OBs (Figure 5A). These three unsaturated fatty acids were also the main fatty acids in the *sn*-1/3 positions. The saturated fatty acids of chia seed OBs were mainly located in the external *sn*-1 and *sn*-3 positions: 10.1 % palmitic acid (16:0) and 4.2 % stearic acid (18:0). These findings are in agreement with those reported for the *sn*-2 fatty acid composition of chia seed oil, where linolenic, linoleic and oleic acids accounted for 63.8%, 25.1% and 6.4%, respectively [46]. 

In camelina seed OBs (Figure 6C), the *sn*-2 position of the TAGs was predominantly occupied by α-linolenic acid (52.6 %; 18:3 *n*-3, ALA), linoleic acid (30.3 %; 18:2 *n*-6, LA) and oleic acid (16.0 %; 18:1 *n*-9, O); representing 99% of the total fatty acids in the *sn*-2 position. The ω6-LA/ω3-ALA ratio in the *sn*-2 position of camelina seed TAGs was 0.58, which is lower than the ω6/ω3 PUFA ratio of 0.78 found for camelina seed OBs (Figure 5A). In the *sn*-1/3 positions, the main fatty acids (>5%) were linolenic acid (29.1%), gondoic acid (21.9%; C20:1 *n*-9), oleic acid (15%), linoleic acid (11.9%) and palmitic acid (8.6%). The erucic acid (22:1 *n*-9) was mainly esterified at the *sn*-1/3 positions of the TAGs (99.5 ± 0.1%) and accounted for 4.7% of the total fatty acids in these external positions. 

Regarding the repartition of each fatty acid in the *sn*-2 or *sn*-1/3 positions of the TAGs, we found that about 35% of the oleic acid was esterified in the *sn*-2 position for both chia and camelina seed OBs (35.6 ± 0.5% vs. 34.9 ± 0.2%, respectively). In the TAGs from chia seed OBs, the relative proportions of linoleic and linolenic acids esterified in the *sn*-2 position were significantly lower (*p* < 0.05) than in the TAGs from camelina seed OBs (18:2 *n*-6: 45.2 ± 0.2% vs. 56.1 ± 0.2%; 18:3 *n*-3: 35.0 ± 0.1 vs. 47.5 ± 0.3%, respectively). The saturated fatty acids, palmitic and stearic acids, were mainly esterified in the *sn*-1/3 positions (>98%) of TAGs in both chia and camelina seed OBs. 

Our results on the stereospecific distribution of the fatty acids in the TAG molecules of chia and camelina seed OBs, mainly their proportion in the central *sn*-2 position, will contribute to a better understanding of the absorption and health benefits of these plant lipid sources rich in PUFA. Our results are in agreement with previous studies reporting that most vegetable oils contain unsaturated fatty acids (oleic, linoleic and linolenic acids) at the *sn*-2 position and saturated fatty acids at the *sn*-1 and/or *sn*-3 positions [61]. 

#### 3.2.4. Plant Sterols, Tocopherols and Phenolic Compounds

**Figure 7:**
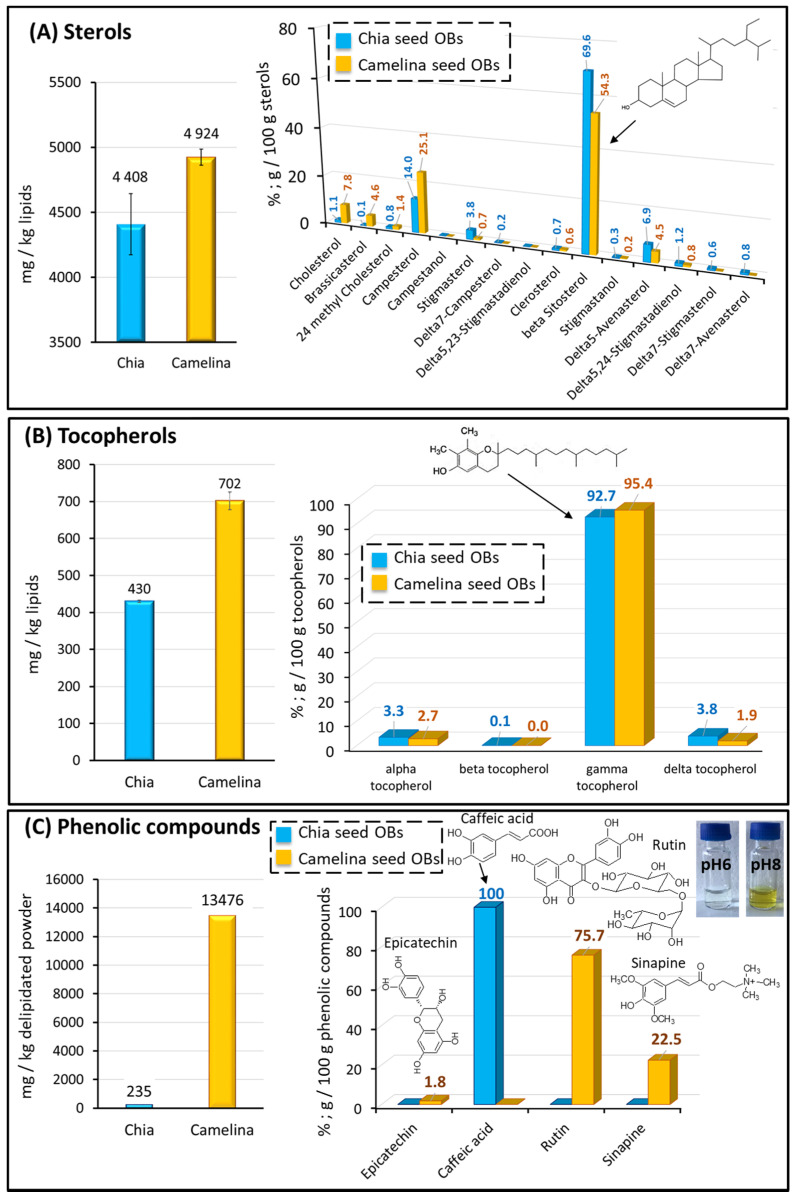
Composition of chia and camelina seed oil bodies: (**A**) sterols, (**B**) tocopherols, (**C**) phenolic compounds, with an illustration showing the yellow colour of rutin at basic pH. The chemical structures of the main components are indicated in the figures.

As a result of their health benefits, the contents in the bioactive minor lipid components from oilseeds have received increasing interest. In this study, the content of lipophilic tocopherols and sterols was determined in the OB-rich cream, and the phenolic compounds were quantified in the delipidated aqueous extracts (Figure 7). The presence of compounds with high antioxidant activity in the aqueous extracts, particularly tocopherols and phenolic compounds, could favour a natural protection against oxidation. 

##### Plant Sterols

Plant sterol compositions of chia seed OBs and camelina seed OBs are presented Figure 7A. Chia seed OBs and camelina seed OBs contained 4408 ± 234 mg/kg and 4924 ± 62 mg/kg of oil, respectively. This is in agreement with the literature, since plant sterols were reported to account for 4132 mg/kg in chia seed oil [9] and a content in the range of 3600–5900 mg/kg in camelina seed oil [20]. These sterol contents found in chia and camelina seed OBs were higher than the amounts reported for hemp seed OBs (3415 mg/kg oil, [1]). β-sitosterol was the main sterol, contributing 69.6 ± 0.2 % of all sterols (3069 mg/kg oil) in chia and 54.3 ± 0.2 % of all sterols (2674 mg/kg oil) in camelina seed OBs. This is a typical distribution of plant sterols, similar to other oilseeds, where β-sitosterol was identified as the main component [1,9,10,20]. Campesterol was the second most common sterol, with 14 % in chia and 25 % in camelina seed OBs, in agreement with previous reports on chia and camelina oils [9,20]. Δ^5^-avenasterol accounted for 6.9 ± 0.1% and 4.5 ± 0.1% in chia and camelina seed OBs, respectively. Camelina seed OBs showed relatively high contents of two unusual sterols, cholesterol (7.8 ± 0.1%) and brassicasterol (4.6 ± 0.1%), in agreement with previous reports on camelina oil [20], while chia seed OBs did not contain these sterols.

This study showed that chia and camelina seed OBs are interesting sources of plant sterols in the human diet, i.e., containing 4.4 and 4.9 g/kg oil, respectively. Chia and camelina seed OBs could therefore contribute to reaching the recommendations for the daily intake of plant sterols (2 g/day; [62]) that are required for the dietary prevention of cardiovascular diseases. 

##### Tocopherols

Tocopherol compositions of chia seed OBs and camelina seed OBs are presented in Figure 7B. The amounts of total tocopherols were 430.4 ± 3.4 mg/kg of oil and 702.2 ± 24.0 mg/kg oil in chia seed OBs and camelina seed OBs, respectively, with a significantly higher (*p* < 0.05) amount in camelina seed OBs. The main isomer was γ-tocopherol, contributing 92.7% (400 mg/kg oil) and 95.4% (670 mg/kg oil) of total sterols in chia and camelina seed OBs, respectively. Chia and camelina seed OBs also contained α-tocopherol (3.7 and 2.7 %, respectively) and δ-tocopherol (3.8 and 1.9 %, respectively). 

##### Phenolic Compounds

The phenolic compounds were analysed in the delipidated aqueous extracts to characterise the antioxidant molecules located in the aqueous phase surrounding the OBs (Figure 7C). The amounts of phenolic compounds were 235 mg/kg in the chia seed aqueous extract and 13476 mg/kg in the camelina seed aqueous extract. In the chia seed aqueous extract, caffeic acid accounted for 100% of the phenolic compounds, in agreement with the high amount in chia seeds reported in previous studies [63,64]. In the camelina seed aqueous extract, epicatechin (flavanol), sinapine (choline ester of sinapic acid) and rutin (flavonol) were identified. The phenolic composition of camelina seed aqueous extracts was dominated by rutin, representing 75.7% of the phenolic compounds and accounting for 10204 mg/kg. Rutin is considered among the top therapeutically active phytochemicals, as recently reviewed [65]. Rutin was responsible for changes in colour from milky white to yellow/brown of the camelina seed extract on increasing the pH from 6.4 (pH of aqueous extract obtained in milliQ water) to 7.5 (Figure 7C). An increase in the pH of the standard rutin solution from pH 6 to pH 8 confirmed the formation of yellow compounds at basic pH (Figure 7C). Condensed tannins were not detected in the chia and camelina seed aqueous extracts. We hypothesised that the condensed tannins remained in the meal during processing and that they were therefore not released in the aqueous extracts, as previously reported [66].

This is the first reported determination of the phenolic compound composition in chia and camelina seed aqueous extracts. Several authors have analysed the camelina phenolic compounds in the seeds, in the oil obtained after cold-pressing of the seeds and in the oilseed cake that is a protein-rich by-product of the deoiling process [67,68,69]. These authors reported that the phenolic compounds were mainly accumulated in the cake and that only minor amounts were recovered in the oil. High amounts of flavonol rutin were found in the cakes obtained from camelina seeds [67,68], while very low amounts were recovered in the oil [69]. This means that the aqueous extraction of camelina seeds performed in this study favours the presence of phenolic compounds such as rutin in the products when compared to oil, which could be interesting for food applications and health benefits. The phenolic compounds present in the chia and camelina seed aqueous extracts may participate in the antioxidant activity towards protein and lipid oxidation [67]. This work supplies new information about the main phenolic compounds present in chia seed and camelina seed aqueous extracts, which are important dietary sources of natural antioxidants for the prevention of diseases caused by oxidative stress. 

In conclusion about the minor bioactive compounds, it seems important to highlight that the high levels of tocopherols and phenolic compounds present in the aqueous extracts could protect lipids and proteins from oxidation, imparting an extended shelf life, as previously reported for these oils [70]. 

### 3.3. Physical Stability of Chia and Camelina Seed OBs as a Function of pH: Toward Innovative and Sustainable Food Applications

In the context of food formulations, which have a range of pH values from neutral to acidic, the third part of this study was dedicated to the physical stability of chia seed and camelina seed OBs as a function of pH. A multiscale approach permitted the recovery of information as a function of pH from the macroscopic level (physical stability of the emulsions containing the OBs) to the microscopic level (microscopy observations) and to the level of the particles by determining the OBs surface properties (ζ-potential measurements).

**Figure 8:**
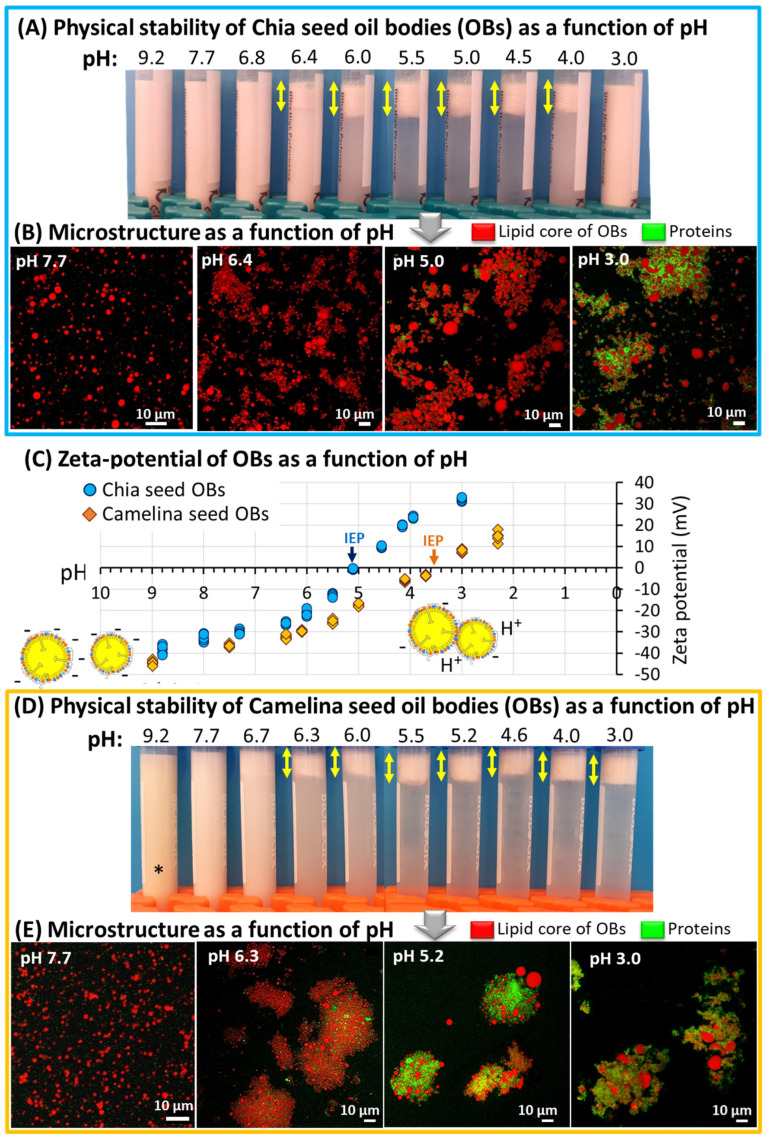
Effect of pH on natural oil-in-water emulsions composed of chia and camelina seed oil bodies (OBs). (**A**,**D**) Physical stability of the emulsions as a function of pH observed at the macroscopic level; (*) indicates the browning of camelina seed aqueous extract at basic pH due to the presence of rutin. (**B**,**E**) Confocal laser scanning microscopy (CLSM) images of the emulsions as a function of pH, the cores of the OBs were stained with Nile red (red colour) and the proteins with Fast Green FCF (green colour) fluorescent dyes. The scale bars are indicated in the figures. (**C**) Zeta-potential of the OBs as a function of pH; illustrations of OBs exhibiting electrostatic repulsions at pH 9 and aggregation near the isoelectric point (IEP).

Figure 8 shows the effect of pH on the physical stability and surface properties of chia and camelina seed OBs. At the macroscopic level, we characterised physical stability of the emulsions containing chia seed OBs (Figure 8A) and camelina seed OBs (Figure 8D) as a function of pH. Decreasing the pH to below pH 6.5 led to physical instability, with phase separation and formation of an OB-rich layer at the top of the containers within 2 h of storage at room temperature (no further evolution after 2 days of storage at 4°C). At the microscopic level, CLSM images revealed individual OBs for pH values above 6.5 and the formation of aggregates below pH 6.5 (Figure 8B,E). For low pH values, CLSM images showed that the proteins corresponding mainly to oleosins located at the surface of the OBs (Figure 3) were involved in the formation of the aggregates. Coalescence of OBs was also observed at low pH values, indicating fusion of natural OBs to form large OBs. The dispersion of chia seed OBs at pH 3 was turbid and did not show any phase separation at the macroscopic level. However, CLSM images showed the presence of both aggregates and free OBs. 

ζ-potential measurements showed changes in the OB surface properties as a function of pH (Figure 8C). The ζ-potential values of chia and camelina seed OBs evolved from negative to positive with decreasing pH. This was interpreted as changes in the charge of their surface components, i.e., the phospholipids (Figure 4) and proteins (oleosins and caleosins; Figure 3F). At pH 9, the ζ-potential values were −38.4 ± 2.1 mV for chia seed OBs and −44.5 ± 1.1 mV for camelina seed OBs. The absolute values of ζ-potential decreased with pH until reaching the isoelectric point (IEP; ζ = 0 mV) at pH 5.1 for chia seed OBs and pH 3.6 for camelina seed OBs. For pHs below the IEP, the ζ-potential values were positive. At pH 3, the ζ-potential values were +32.0 ± 0.8 mV for chia seed OBs and +8.0 ± 0.8 mV for camelina seed OBs. The differences observed between chia and camelina seed OBs were attributed to differences in their surface composition, i.e., the proteins, the phospholipids or both. The trends in ζ-potential values measured in this study as a function of pH for chia seed OBs and camelina seed OBs are in agreement with the literature for maize OBs, sesame OBs, peanut OBs, sunflower OBs, soybean OBs and hemp seed OBs [1,23,24,46]. The IEPs measured in this study were also in agreement with the literature, i.e., in the range of pH 3.5 to pH 5 [1,23,24,46]. 

This study showed the importance of pH on the physical stability of emulsions containing natural OBs. A multiscale approach permitted the understanding of the physical instability as a function of pH of the emulsions containing the OBs, and showed that the surface composition and properties of the OBs govern their physical stability. Decreasing the pH (increasing the amount of H^+^ cations) altered the physical stability of the natural oil-in-water emulsions by inducing aggregation of OBs as a result of the decrease in the electrostatic repulsions between OBs associated with low negative ζ-potential values. The formation of large aggregates of OBs favours their phase separation from the aqueous phase and as a result, they rose to the top of the containers (due to the low density of lipid-rich OBs). 

These results show the impact of pH on the physical stability of chia seed and camelina seed OBs, with the formation of aggregates of OBs near the IEP, and are in agreement with the literature on other sources of OBs [1,6,23]. Such physical instability of OBs as a function of pH should be considered for the development of food products such as fermented food products. 

## 4. Conclusions

In this study, the biodiversity of oilseeds was explored and the microstructure, composition and physical stability of chia and camelina seed OBs were analysed. The chia and camelina seed OBs were recovered from seeds using a green refinery process including ultrasonication, grinding and centrifugation. This study showed that chia and camelina oilseeds are interesting plant resources for the extraction of OBs and utilisation in innovative food applications due to (i) their high amount of lipids (35% and 42%, respectively) that are naturally dispersed as physically and chemically stable OBs of micron size and (ii) their high content of ω3 fatty acids (ALA: 64% and 30% of the total fatty acid content, respectively), low ω6/ω3 PUFA ratios, phospholipids (0.23 and 0.26%, respectively) and other bioactive compounds (plant sterols, tocopherols and phenolic compounds). The comparative analysis performed between chia and camelina seed OBs highlighted differences due to biodiversity. In the near future, the biodiversity of plant sources (oilseeds, nuts) should be further valorised to provide lipids and proteins of interest in the human diet. Chia and camelina seeds represent an opportunity to satisfy (i) the increasing demand for sustainable seed productions, (ii) the necessity to diversify plant sources to feed the rising worldwide population and (iii) the use of aqueous extracts and OBs for high-quality plant-based food products providing health benefits for consumers. Aqueous extracts produced from chia and camelina seeds as well as their OBs should be considered valuable plant resources for the food industry to prepare natural oil-in-water emulsions, and for consumers to reduce the ω6/ω3 PUFA ratio in the diet by increasing daily intakes in ω3 PUFA. 

## Data Availability

The datasets used and/or analysed during the current study are available from the corresponding author on request.
